# Excess primary healthcare consultations in Norway in 2024 compared to pre-COVID-19-pandemic baseline trends

**DOI:** 10.1186/s13690-025-01817-8

**Published:** 2026-01-02

**Authors:** Richard Aubrey White, Beatriz Valcarcel Salamanca, Aslaug Angelsen, Dinastry Pramadita Zakiudin, Aristomo Andries, Gunhild Alvik Nyborg

**Affiliations:** 1 Norwegian Institute of Public Health, Oslo, Norway; 2 Norwegian University of Science and Technology, Trondheim, Norway; 3 Oslo University Hospital, Oslo, Norway

**Keywords:** COVID-19, Primary healthcare, Long COVID, PASC, Post-acute sequelae of COVID-19, PCC, Post COVID-19 condition, Norway, Health policy

## Abstract

**Background:**

The risk of post-acute sequelae of COVID-19 (PASC) is estimated at 3–6% per infection in 2024. We hypothesized that widespread SARS-CoV-2 infections could lead to population-level consequences. Our previous study identified substantial increases in Norwegian primary healthcare consultations in 2023—compared to pre-pandemic levels—for conditions associated with acute COVID-19 and PASC. This study extended that analysis to 2024. We then assessed whether observed patterns were compatible with our hypothesis.

**Methods:**

We used data from the Norwegian Syndromic Surveillance System, which captures nationwide primary healthcare consultations for 102 ICPC-2 codes (out of a possible 710) that are relevant for infectious disease surveillance and some post-acute infection syndromes. Bayesian linear regression models were fitted to 2010–2019 trends, adjusting for population changes, to estimate expected values for 2024. Excess consultations were calculated by age and sex. A COVID-19 community spread was proxied by vaccination-adjusted weekly hospitalization rates.

**Results:**

In 2024, there were 17,800,365 consultations, corresponding to an absolute excess of 1,185,231 consultations, or a 7.1% relative excess, compared to the modelled baseline. The 10 code combinations with largest absolute excess in 2024 were respiratory infections (325,726 excess consultations; 20% relative excess), fatigue (205,381; 70%), psychological symptom/complaint other (188,978; 87%), acute stress reaction (182,079; 76%), feeling depressed (126,783; 133%), hyperkinetic disorder (112,763; 116%), abdominal pain/cramps general (84,544; 29%), memory disturbance (39,177; 63%), conjunctivitis (34,643; 59%), and infectious disease other/NOS (33,556; 81%). COVID-19 community spread showed the strongest correlations with conjunctivitis, strep throat, respiratory infections as a group (R**), fatigue, infectious disease other, memory disturbances, and pneumonia. Deviations from pre-pandemic trends varied: respiratory and psychological disorders worsened from 2020 onward and several conditions showed dramatic excess from 2022–2024. Females 15–29, children, adolescents, and young adults had disproportionately large relative excesses for consultations for memory disturbances.

**Conclusions:**

Primary healthcare consultations in 2024 significantly exceeded pre-pandemic expectations, especially for conditions linked to acute COVID-19 and PASC, though the two cannot be differentiated in these data. While other factors undoubtedly also play a role, findings are compatible with ongoing population-level health impacts associated with repeated SARS-CoV-2 infections, particularly among women, children, adolescents, and young adults. These results emerged under a national COVID-19 strategy that does not account for post-acute consequences of SARS-CoV-2 infection.

**Supplementary Information:**

The online version contains supplementary material available at 10.1186/s13690-025-01817-8.

**Table:** 

Text box 1. Contributions to literature	
• Since 2022, the Norwegian population has been repeatedly infected with SARS-CoV-2. • There has been a substantial increase in national healthcare utilization from 2020 to 2024—higher than pre-pandemic expectations. • Many of the conditions with the highest healthcare utilization increases are associated with acute and post-acute COVID-19 sequelae. • Women (a group known to be at higher risk of post-acute COVID-19 sequelae) and children and adolescents (a group often excluded from long COVID policy considerations, including vaccine recommendations) have disproportionately higher healthcare utilization than expected for some syndromes that are associated with acute and post-acute COVID-19 sequelae.	

## Background

The COVID-19 pandemic has altered global healthcare utilization patterns, with impacts extending far beyond the acute phase of infection. Post-acute sequelae of COVID-19 (PASC) is now recognized as a systemic, multi-organ disorder, often causing prolonged fatigue, cognitive and neurological dysfunction, cardiovascular complications, and other disabling symptoms [1]. The burden of PASC is substantial [1–4]; an estimated 400 million individuals require support globally [5], with estimates attributing an annual economic loss of approximately 0.5% of gross domestic product [6, 7].

Given this substantial burden, countries have adopted varying approaches to managing ongoing COVID-19 transmission. Norway’s current COVID-19 strategy [8], implemented since 2022, differs notably from World Health Organization and other international guidelines [9] and is dependent upon the Norwegian population being repeatedly reinfected with SARS-CoV-2. The Norwegian Institute of Public Health (NIPH) outlines the rationale behind Norway’s COVID-19 strategy:

“It is now mainly population immunity that is keeping the epidemic in check … Stronger measures to limit the spread of infection have two important disadvantages. Firstly, the measures can be resource-intensive, restrict freedoms and weaken the economy and perhaps public health. Secondly, maintaining population immunity depends on the virus circulating in the population” [10].

This strategy leans on repeated SARS-CoV-2 infections to maintain population immunity and less on booster vaccinations. Since 2023, the general population has been advised not to get tested for COVID-19 if experiencing symptoms of respiratory tract infections [11]. This creates conditions where repeated SARS-CoV-2 reinfections are common across the population. Although reinfection increases cumulative PASC risk [12–14], the Norwegian government's COVID-19 strategy [8] and NIPH’s risk assessment of the strategy focused exclusively on acute consequences, disregarding the potential negative impact on public health from PASC [10]. Elsewhere, NIPH has claimed that frequent reinfection by SARS-CoV-2 is beneficial for reducing the risk of PASC [15], a view that contrasts with prevailing scientific consensus [16].

Understanding the ongoing risk of PASC is crucial for interpreting healthcare utilization. A recent Norwegian study estimated a 6% PASC risk for triple-vaccinated individuals after a first Omicron infection [17], aligning with international findings [18, 19]. Results on reinfection are mixed: a UK study found 28% lower odds of PASC after a second versus first infection [12], while U.S. and Canadian data show consistent risk across multiple infections [13, 14]. Conservative estimates suggest a current per-infection PASC risk of 3–6% in Norway, implying substantial cumulative burden over time.

However, the detection of this burden in routine healthcare data is complicated by Norway’s use of the International Classification of Primary Care, 2nd edition (ICPC-2) diagnosis coding system in primary care [20]. ICPC-2 codes capture the reason for the patient’s encounter or visit to the healthcare provider; approximately half represent diagnoses and half represent symptoms [20, 21]. Unlike the International Classification of Diseases (ICD) system, it has not been updated with a specific diagnosis code for PASC. As a result, general practitioners who suspect that their patients suffer from PASC must instead select a diagnosis based on the most dominant symptom, such as fatigue or memory loss [22]. This coding limitation may obscure the syndromic nature of PASC and disperse its manifestations across diagnostic categories, complicating efforts to quantify its true impact.

Our previous study found that nationwide sick leave and primary healthcare consultations in 2023 exceeded expected trends for conditions consistent with both acute COVID-19 and PASC [23]. This study extends that nationwide analysis to 2024, identifying diagnostic code combinations with the largest absolute deviations from 2010–2019 modelled baselines, with age- and sex-specific results. We then assessed whether these deviations plausibly reflected population-level consequences of widespread repeated SARS-CoV-2 infections.

These findings provide crucial data in the Norwegian context, where the national COVID-19 strategy emphasizes the assumed benefits of sustaining population immunity through repeated SARS-CoV-2 infections, contrary to available evidence on PASC. However, given the global endemicity of SARS-CoV-2, the results also hold broader international relevance [24].

## Methods

### Data sources

The Norwegian Syndromic Surveillance System (NorSySS; Norwegian: *Det norske syndromiske overvåkingssystemet*) is a nationwide public health surveillance system designed to detect outbreaks of infectious diseases and provide early warning for implementation of necessary control measures [25]. NorSySS surveils the number of consultations at general practitioners and out-of-hours primary care facilities [23]. NorSySS' data source is KUHR (Control and Payment of Health Reimbursements; Norwegian: *Kontroll og utbetaling av helserefusjoner*), which is a system that manages reimbursement claims from healthcare providers and institutions to the state in Norway. The system is owned by the Norwegian Directorate of Health. KUHR is a system within KPR (Municipal Patient and User Register; Norwegian: *Kommunalt pasient- og brukerregister*) that contains data from municipalities about individuals who have applied for, receive, or have received health and care services. While some individuals may choose private healthcare over their publicly subsidized primary healthcare physician, less than 4% of the Norwegian population lack access to a publicly subsidized permanent primary healthcare physician (the data source for NorSySS) [26]. This system is the foundation of the primary health care system in Norway [27]. The data source is routinely audited to ensure no fraudulent billing is occurring [28].

NorSySS does not have full coverage of the 710 ICPC-2 codes available to primary healthcare physicians. Rather, NorSySS has access to 102 ICPC-2 codes that are relevant for infectious disease surveillance and some post-acute infection syndromes, which aligns with its surveillance purpose. NorSySS covers the following categories: general and unspecified (13/53); blood, blood forming organs and immune mechanism (4/25); digestive (15/67); eye (2/35); ear (6/28); cardiovascular (0/41); musculoskeletal (0/53); neurological (1/37); psychological (5/43); respiratory (31/54); skin (16/58); endocrine/metabolic and nutritional (0/31); urological (1/29); pregnancy, childbearing, family planning (0/40); female genital (4/53), male genital (4/36), social problems (0/27). Consultations for the missing ICPC-2 codes are registered in NorSySS under the ICPC-2 code “XXX”.

We extracted one outcome measure from NorSySS: The number of primary healthcare consultations per ICPC-2 code combination. A consultation was defined as one interaction with a primary healthcare practitioner that corresponds to one of the following: home visit by a general practitioner (day/night), consultation with a general practitioner (day/night), consultation for being called to the office for immediate help of a patient, e-consultation with a general practitioner and/or emergency room (day/night). These correspond to the KUHR tariff codes 11ad, 11ak, 2ad, 2ak, 2fk, 2ae, 2aek, 2aef.

The extracted data was age- and sex-specific for age groups 5–14, 15–19, 20–29, 30–64, and 65 + for males and females.

### Composite ICPC-2 code combinations

Composite ICPC-2 code combinations were extracted directly from NorSySS. In addition to single ICPC-2 codes (e.g., A04), NorSySS includes two predefined composite groupings: gastroenteritis (D11 vomiting, D70 gastrointestinal infection, and D73 gastroenteritis presumed infection), and R** respiratory infections (R01, R02, R03, R04, R05, R07, R08, R09, R21, R24, R25, R27, R29, R33, R71, R72, R74, R75, R76, R77, R78, R79, R80, R81, R82, R83, R99, R991, and R992). Internally at NIPH, the R** code combination is used as a proxy for respiratory infections. Descriptions of the R codes are available in Additional file 1.

These groupings were previously developed by epidemiologists at NIPH and have been used in routine surveillance for many years. No additional groupings were created in NorSySS or in this analysis, as the system contains only a subset of all possible ICPC-2 codes. Constructing broader diagnostic groupings would therefore not have been appropriate, as they would be incomplete and potentially misleading.

### Comparing 2024 against a 2010–2019 baseline

Data was extracted for 99 ICPC-2 codes, and 2 ICPC-2 code combinations (Additional file 1), representing the number of primary healthcare consultations per year. While NorSySS contains 102 ICPC-2 codes, the codes R991 (probable/suspected COVID-19) and R992 (confirmed COVID-19) were not included individually, as they did not exist in NorSySS prior to 2020, and therefore a 2010–2019 baseline cannot be estimated. R991 and R992 are rather included in the R** code combination. A73 malaria was excluded due to insufficient cases.

We used the data from 2010–2019 to predict expected baselines for 2024, then calculated the excess values for 2024 by subtracting the observed values from the expected baselines.

To calculate the expected/excess values for 2024, one analysis was performed for each ICPC-2 code combination.

To investigate the appropriate model for the expected baseline, three linear regressions were performed on data between 2010–2019:

- Model 1:
  - Outcome: Rate/100k
  - Covariate: Year as a continuous linear variable with three-way interactions with age and sex.
- Model 2:
  - Outcome: Rate/100k
  - Covariate: Year as a cubic spline with two degrees of freedom with three-way interactions with age and sex.
- Model 3:
  - Outcome: Rate/100k
  - Covariate: Year as a cubic spline with three degrees of freedom with three-way interactions with age and sex.

The model with the lowest Akaike Information Criterion (AIC) was selected, and then a Bayesian linear regression was performed using the selected model between 2010–2019, with 4 chains each containing 20,000 iterations. To account for heteroscedasticity, residual standard deviation was allowed to vary by age strata. Model fit was assessed via residuals. For ICPC-2 codes R78 and P03 the residual fit was poor, so the automatic model selection process via AIC was disregarded and year as a continuous linear variable was manually selected. The Bayesian linear regression was implemented using the “brms” package in R, which uses gradient-based Markov chain Monte Carlo algorithms [29–31]. The expected baseline for 2020 to 2024 was then calculated by estimating the posterior of the rate/100 k. The model results were aggregated up to calculate totals for all ages and all sexes. The expected baselines were then used to calculate the excess values and corresponding 95% prediction intervals (95% PI).

The excess values were then restricted to 2024 and corrected for multiple testing using false discovery rates (FDR) with a threshold of 0.05. The ten ICPC-2 code combinations with the largest absolute excesses were then considered for further closer investigation.

### Temporal association between ICPC-2 diagnoses/symptoms and community spread of COVID-19 between 2020 and 2024

As described in our earlier study, there is no consistent data on community spread of COVID-19 in Norway for the entire period of 2020 to 2023. Polymerase chain reaction (PCR) testing and registering of test results were only reliable until the implementation of the “vaccine-only strategy” in 2022, and wastewater ribonucleic acid (RNA) concentration measurements for SARS-CoV-2 were in place since mid-2022, however these measurements were discontinued late 2023 [23].

A proxy therefore had to be created to identify a temporal correlation with community spread of COVID-19: the “community spread index”. Specifically, we constructed a weekly incidence of COVID-19 hospitalizations in a hypothetical population where no-one is vaccinated, to remove the period effect of pre/post-vaccinated Norway, in the same manner of our previous study [23].

As NIPH ceased reporting the number of patients hospitalized with SARS-CoV-2 in week 25 of 2024, we needed to impute the data between weeks 26 and 52 in 2024. This was done by fitting a linear regression during the period 2020-W14 to 2024-W25, with the outcome being the weekly incidence of COVID-19 hospitalizations in a hypothetical population where no one is vaccinated, and the explanatory variables the proportion of lab positive SARS-CoV-2 (as reported to MSIS laboratory database) interacting with the proportion of primary healthcare consultations for COVID-19 (as reported to NorSySS), both expressed as cubic splines with two degrees of freedom. The remaining data for 2024 (2024-W26 to 2024-W52) was predicted using this fitted model. This variable was then rescaled into a maximum of 1 and a minimum of 0 for interpretation purposes, then summed over quarterly periods (i.e. weeks 1–13, 14–26, 27–39, 40–52).

For each ICPC-2 code combination, the number of consultations per quarterly period were calculated and Pearson’s correlation coefficients were then calculated on these quarterly datasets for:

1. Number of primary healthcare consultations versus community spread of COVID-19 in the same quarter.
2. Number of primary healthcare consultations versus community spread of COVID-19 in the previous quarter.
3. Number of primary healthcare consultations versus community spread of COVID-19 two quarters prior.

Pearson's correlation coefficients with significance levels under 5% were displayed in the main figures, without correcting for multiple testing, as they were not utilized as standalone analyses. Rather, they were utilized as descriptive aids to help in the interpretation of the main results—excess/deficit in 2024 versus pre-pandemic trends. We note that these temporal analyses are based on *N* = 20 data points (5 years × 4 quarters), which inherently limits statistical power for detecting correlations.

Numerous human respiratory viruses demonstrate pronounced winter seasonality in temperate climates, however, animal models suggest that environmental conditions may not translate to measurable impacts on SARS-CoV-2 transmission [32]. US data has identified possible bimodal seasonality for SARS-CoV-2, likely linked to viral evolution and cyclical diversity [33]. Other models show varying results [34]. However, in our Norwegian data, we did not observe any seasonality, with no month from 2020–2024 demonstrating consistent patterns for more than 3 years (Additional file 2). Even when only considering 2023 and 2024, the monthly patterns were not consistent (Additional file 2), and as such, we did not account for seasonality in our analyses.

### Statistical software

All analyses were performed in R, version 4.5.1 [35], using the *org* [36] and *plnr* [37] packages as the core of the analytical workflow.

## Results

### Total number of primary healthcare consultations

**Fig. 1:**
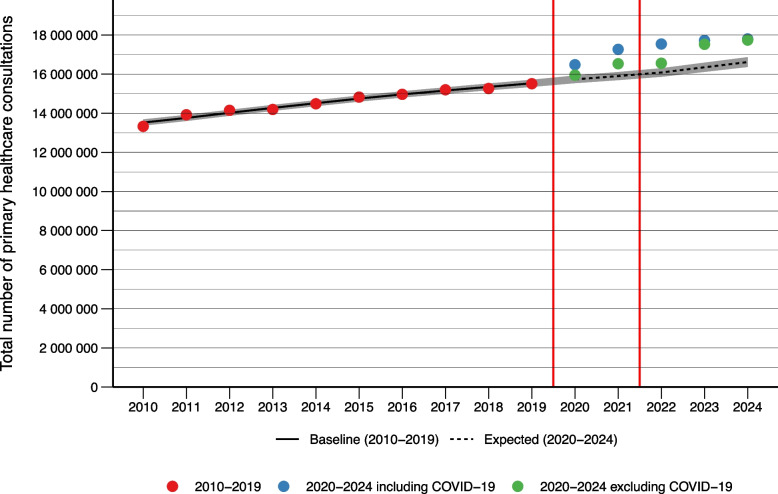
Total number of primary healthcare consultations per year from 2010–2024, with an expected baseline calculated using data from 2010–2019

Considering a baseline modelled on 2010 to 2019, it was expected that there would be 16,615,134 (95% PI: 16,362,412 to 16,868,392) total consultations in 2024. We observed 17,800,365, an absolute excess of 1,185,231 (95% PI: 931,973 to 1,437,953), corresponding to a relative excess of 7.1% (95% PI: 5.5% to 8.8%) (Fig. 1).

### Primary healthcare consultations for COVID-19

The number of primary healthcare consultations for COVID-19 has changed dramatically since 2020; reaching a new low in 2024; 6% of the 2022 peak (Table 1).

**Table 1:** Number of primary healthcare consultations for COVID-19 (R991 + R992) per year compared to the total number of excess consultations (as defined by comparing against the modelled 2010–2019 baseline)

Year	Number of excess consultations (compared to modelled 2010–2019 baseline)	Number of primary healthcare consultations for COVID-19 (R991 + R992)	Percentage of excess consultations directly explained by COVID-19 (R991 + R992)
2020	749,884 (554,273 to 942,100)	525,595	70.1% (55.8% to 94.8%)
2021	1,365,511 (1,159,907 to 1,572,100)	735,126	53.8% (46.8% to 63.4%)
2022	1,455,987 (1,238,733 to 1,675,021)	982,544	67.5% (58.7% to 79.3%)
2023	1,371,797 (1,135,776 to 1,605,430)	196,785	14.3% (17.3% to 12.3%)
2024	1,185,231 (931,973 to 1,437,953)	60,750	5.1% (4.2% to 6.5%)

### Community spread of COVID-19

The linear regression model used for imputation (fitted on 2020-W14 to 2024-W25 data, predicting 2024-W26 to 2024-W52) showed a high R-squared value of 0.87 for predicting COVID-19 hospitalizations from laboratory positivity rates and primary care consultations. When observing the community spread index, we observed minimal SARS-CoV-2 spread in 2020 and 2021 (Additional file 3). 2022 was defined by three short and intense COVID-19 waves, while 2023 and 2024 were characterized by long periods of medium levels of SARS-CoV-2 transmission (Additional file 3). The total area under the curve for the community spread index for the years 2020 through to 2024 were 0.3, 3.7, 22.9, 13.3, and 6.9 respectively.

### 2024 compared to 2010–2019

**Fig. 2:**
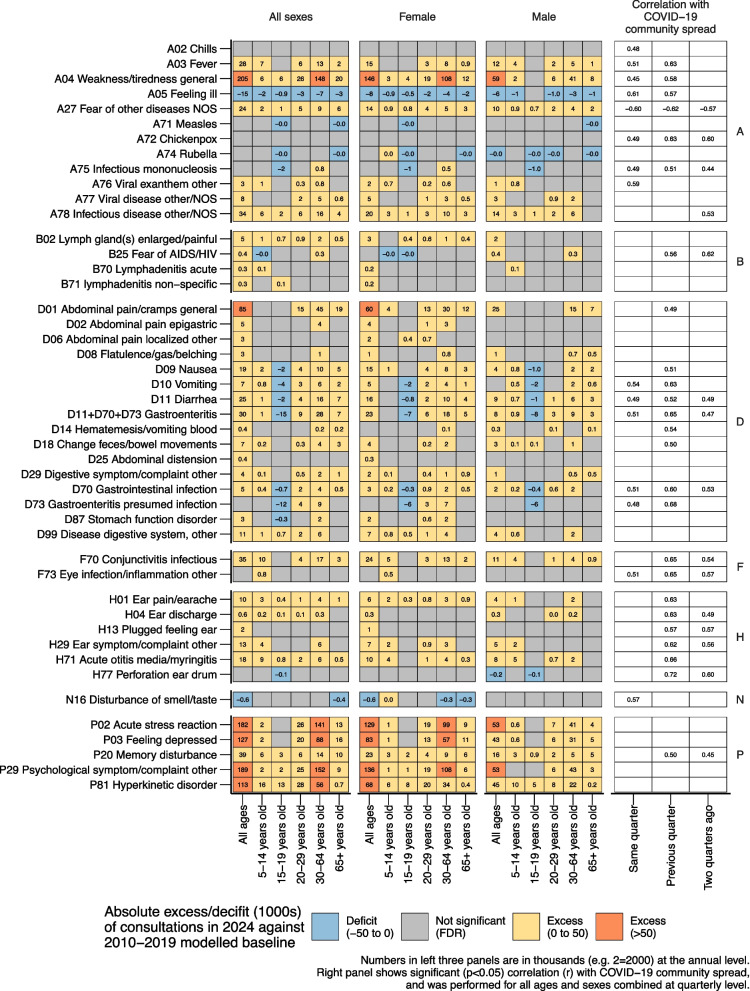
Absolute excess/deficit (in 1000 s) of consultations in 2024 against 2010–2019 modelled baseline and correlation within COVID-19 community spread for ICPC-2 codes A*, B*, D*, F*, H*, N*, P*

**Fig. 3:**
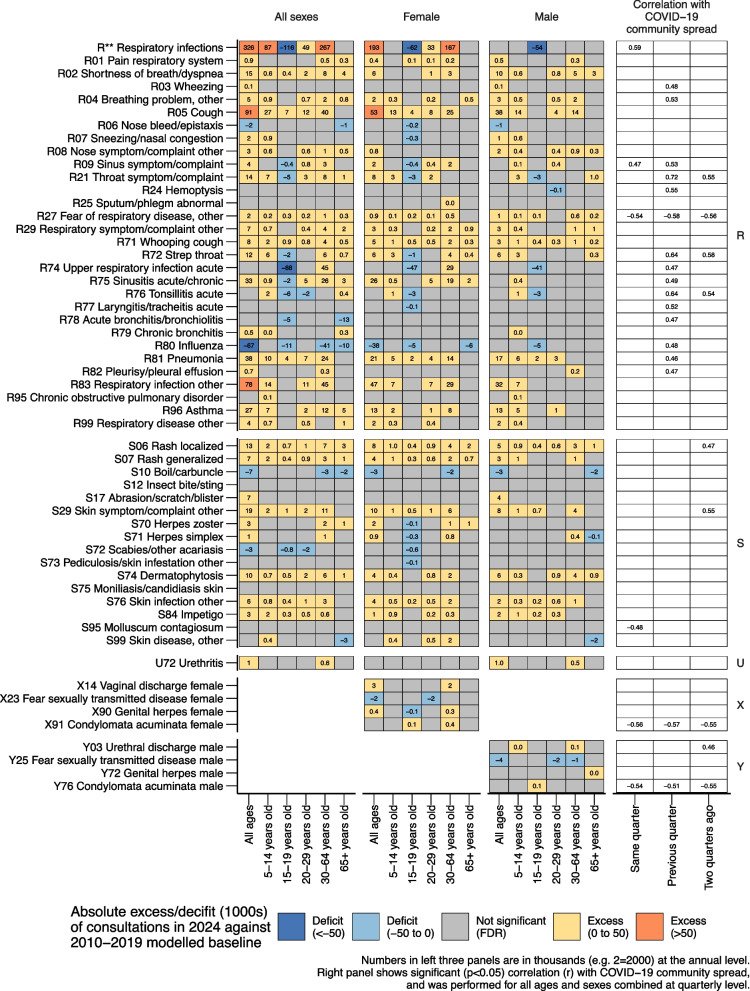
Absolute excess/deficit (in 1000 s) of consultations in 2024 against 2010–2019 modelled baseline and correlation within COVID-19 community spread for ICPC-2 codes R*, S*, U*, X*, Y*

Results for all ages and sexes are available in Figs. 2 and 3 and Additional files 1, 4 and 5.

Examination of the residuals did not reveal any statistical issues with the models (Additional file 5).

**Fig. 4:**
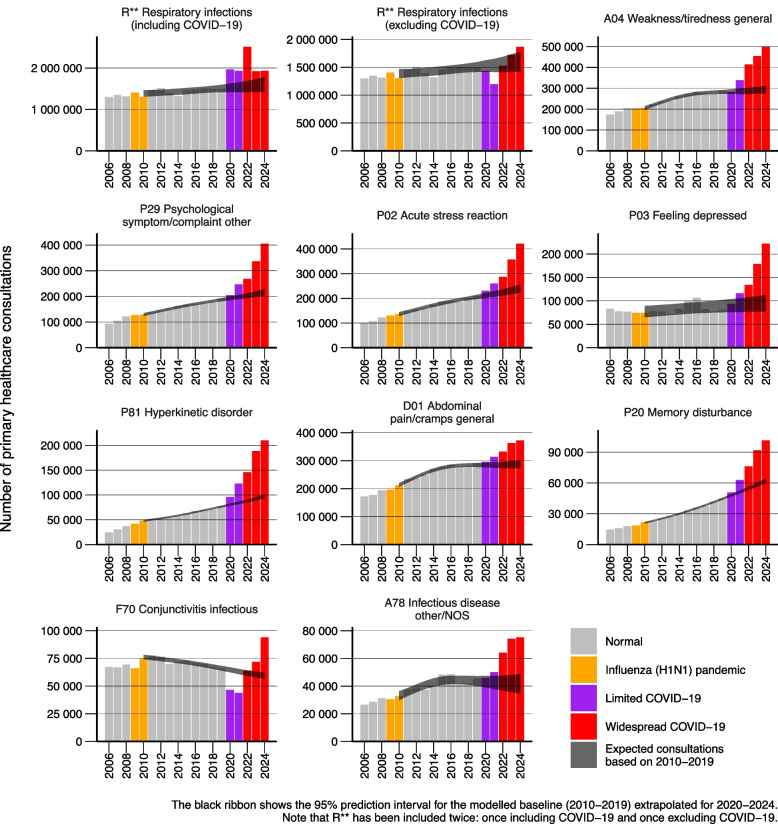
Number of primary healthcare consultations per year from 2006–2024 for the 10 ICPC-2 code combinations with largest absolute excess in 2024 when compared against the 2010–2019 modelled baseline

The ten ICPC-2 code combinations with the largest absolute excesses above the modelled baseline were R** (respiratory infections), A04 (weakness/tiredness general), P29 (psychological symptom/complaint other), P02 (acute stress reaction), P03 (feeling depressed), P81 (hyperkinetic disorder), D01 (abdominal pain/cramps general), P20 (memory disturbance), F70 (conjunctivitis infectious), and A78 (infectious disease other/NOS) (Figs. 2 and 3). For the total population, the absolute excesses remained statistically significant after false discovery rate corrections for all ten conditions (Figs. 2 and 3). We observed no large trend deviations in these ten conditions during or after the 2009/2010 influenza (H1N1) pandemic (Fig. 4).

The ten conditions with the highest absolute excess accounted for 1,333,630 excess consultations in 2024, which exceeded the total observed absolute excess of 1,185,231 consultations. Consultations for acute COVID-19 accounted for 70.1% of total excess in 2020, 53.8% in 2021, 67.5% in 2022, but only 14.3% in 2023 and 5.1% in 2024 (Table 1).

The respiratory infections proxy (R**) represented the largest category of excess consultations in 2024, with an absolute excess of 325,726 consultations (95% PI: 144,288 to 506,189) and a relative excess of 20% (95% PI: 8% to 36%) (Fig. 3, S4 and Table S1). It should be noted that from 2020 onwards, R** includes COVID-19. Within this composite, several individual conditions showed striking increases. R71 (whooping cough/pertussis) had an absolute excess of 8,074 consultations (95% PI: 7,409 to 8,705) and a relative excess of 343% (95% PI: 246% to 506%), representing the highest relative excess of any condition examined (Figs. 3, S4). R81 (pneumonia) had an absolute excess of 38,370 consultations (95% PI: 19,793 to 56,763) and an overall relative excess of 41% (95% PI: 18% to 76%), but showed extreme age-specific variation with children aged 5–14 demonstrating a relative excess of 1,066% (95% PI: 135% to 534,800%) while adults over 65 were not significantly different from the modelled baseline (Table S72). R05 (cough) had an absolute excess of 91,342 consultations (95% PI: 72,401 to 110,361) and a relative excess of 53% (95% PI: 38% to 73%), with children aged 5–14 showing a relative excess of 257% (Figs. 3 and S4 and Table S1). R83 (respiratory infection other) showed an absolute excess of 78,382 consultations (95% PI: 41,318 to 112,734) and a relative excess of 73%. R72 (strep throat) had an absolute excess of 11,935 consultations (95% PI: 7,171 to 16,692) and a relative excess of 41%, with consistent patterns of highest relative excess in school-aged children across respiratory conditions (Figs. 3 and S4 and Table S1).

Mental health presentations showed profound increases across multiple domains. P29 (psychological symptom/complaint other) had an absolute excess of 188,978 consultations (95% PI: 176,676 to 201,509) and a relative excess of 87% (95% PI: 77% to 99%), while P02 (acute stress reaction) showed an absolute excess of 182,079 consultations (95% PI: 165,064 to 198,907) and a relative excess of 76% (95% PI: 64% to 89%). P03 (feeling depressed) had an absolute excess of 126,783 consultations (95% PI: 109,110 to 144,499) and a relative excess of 133% (95% PI: 97% to 187%) (Figs. 2 and S3 and Table S1).

Cognitive and behavioral conditions also showed substantial increases. P81 (hyperkinetic disorder) showed an absolute excess of 112,763 consultations (95% PI: 108,974 to 116,531) and a relative excess of 116% (95% PI: 108% to 125%), with notable gender disparity where females had 2 and 3 times higher relative excess than males. P20 (memory disturbance) showed an absolute excess of 39,177 consultations (95% PI: 36,988 to 41,378) and a relative excess of 63% (95% PI: 58% to 69%), with the highest relative excess in children aged 5–14 (174%), females 20–29 (173%), and females 15–19 (146%) (Fig. 2 and S3 and Table S1).

Other conditions among the top ten absolute excess deviations included A04 (weakness/tiredness general) with an absolute excess of 205,381 consultations (95% PI: 186,161 to 224,576) and a relative excess of 70% (95% PI: 60% to 82%). D01 (abdominal pain/cramps general) had an absolute excess of 84,544 consultations (95% PI: 69,965 to 99,041) and a relative excess of 29% (95% PI: 23% to 36%). F70 (conjunctivitis) showed an absolute excess of 34,643 consultations (95% PI: 31,799 to 37,490) and a relative excess of 59% (95% PI: 51% to 67%), while A78 (infectious disease other/NOS) had an absolute excess of 33,556 consultations (95% PI: 26,397 to 40,606) and a relative excess of 81% (95% PI: 54% to 118%) (Fig. 2 and S3 and Table S1).

Across conditions, consistent age-related patterns emerged, with children aged 5–14 typically showing the highest relative excess, while adolescents aged 15–19 often demonstrated lower or non-significant excess. COVID-19 community spread showed the strongest correlations with conjunctivitis (0.65; 95% CI: 0.29 to 0.85; prior quarter), strep throat (0.64; 95% CI: 0.27 to 0.84; prior quarter), respiratory infections as a group (0.59; 95% CI: 0.20 to 0.82; same quarter), fatigue (0.58; 95% CI: 0.19 to 0.82; prior quarter), infectious disease other (0.53; 95% CI: 0.12 to 0.79; two quarters prior), memory disturbances (0.50; 95% CI: 0.08 to 0.77; prior quarter), and pneumonia (0.46; 95% CI: 0.03 to 0.75; prior quarter). More detailed descriptions—including non-significant correlations—are available in Additional file 6.

Deviations from expected pre-pandemic trends varied in timing across conditions (Fig. 4). R** (respiratory infections, including COVID-19) began deviating in 2020, peaked with larger excess in 2022, then showed lower but still elevated excess in 2023 and 2024. P29 (psychological symptoms), P02 (acute stress reactions), P81 (hyperkinetic disorder), and P20 (memory disturbances) began deviating in 2020, with dramatic worsening from 2022–2024. A04 (weakness/tiredness general), P03 (feeling depressed), D01 (abdominal pain), and A78 (infectious disease other) showed initial deviations beginning in 2021, with A04 (weakness/tiredness general) and A78 (infectious disease other) showing dramatic worsening from 2022 onwards, while P03 (feeling depressed) worsened dramatically from 2023 to 2024. F70 (conjunctivitis) showed a distinct pattern with consultations lower than expected in 2020–2021, returning to expected levels in 2022, then exceeding expectations in 2023–2024.

## Discussion

Primary healthcare consultations in Norway exceeded pre-pandemic expectations by 1,185,231 visits (7.1% relative excess) in 2024. The largest absolute increases were observed for respiratory infections, fatigue, psychological complaints, acute stress reactions, feeling depressed, memory disturbances, and hyperkinetic disorders. The absolute excess of these conditions accounted for 1,333,630 excess consultations, which exceeded the total observed excess of 1,185,231, indicating we captured the most significant increases. Females 15–29, children, adolescents, and young adults observed disproportionately large relative excesses of consultations for memory disturbances. COVID-19 community spread was most strongly correlated with conjunctivitis, strep throat, respiratory infections, fatigue, infectious disease other, memory disturbances, and pneumonia. Deviations from pre-pandemic trends varied across conditions, with respiratory and psychological disorders worsening from 2020 onward and several other conditions showing dramatic excess from 2022–2024.

This is an observational study of aggregated temporal trends, so causal attribution is not possible. However, given that the national COVID-19 strategy is predicated on repeated SARS-CoV-2 reinfections, and that official risk assessments do not account for PASC, this unique situation raises concerns about the long-term health impacts of sustained SARS-CoV-2 circulation at the population level. In the following we therefore examine whether existing literature is compatible with the hypothesis that excess primary healthcare consultations in 2024 could be partially attributed to PASC or alternative explanations.

### Clinical perspectives and diagnostic coding in Norway

Internationally, PASC is recognized as a multi-organ disorder with serious functional consequences [16]. However, prevailing perspectives within Norwegian healthcare differ substantially from this international consensus. In Norway, PASC is often framed as a psychosocial condition—driven more by anxiety and perception than by persistent physiological damage [38–40]. The Oslo Chronic Fatigue Consortium—a nationally influential group of mostly Norwegian clinicians and researchers—has described PASC and other post-infectious disorders as “likely to reflect the brain's response to a range of biological, psychological, and social factors, rather than a specific disease process” [41]. The psychologicalization of PASC is reflected in official Norwegian guidance. For example, the National Health Library (offered as a basic resource package to all healthcare personnel in Norway) lists the following under PASC treatment for patients under 18 years old:

- Psychoeducation: This involves reassuring patients that there is no ongoing viral infection in the body, that alternative causes of the symptoms have been adequately investigated, and that many physical complaints (such as "brain fog", palpitations, shortness of breath, etc.) can be explained by persistent physical stress responses. It is also explained that excessive focus on these symptoms and the expectation that they will occur can have a self-reinforcing effect by increasing stress responses and making the physical complaints more severe. The aim of psychoeducation is therefore to reduce the patient's level of worry and the tendency to focus too much attention on symptoms [42].

These perspectives intersect with structural limitations in diagnostic coding. ICPC-2, used by primary healthcare in Norway, does not include a specific code for PASC. Clinicians are instead required to code the patient’s most dominant symptom. In July 2023, the Norwegian Directorate of Health issued guidance to “dual code” both the most dominant symptom along with R992 (confirmed COVID-19) to flag PASC [22]. However, this practice appears uncommon: in 2024, only 2,884 of the 498,734 consultations for fatigue were dual-coded with R992. This suggests that the guidance has seen limited uptake in practice. Whether this reflects low awareness, limited clinical suspicion, prevailing beliefs that PASC is rare or primarily psychosocial in origin, or something else, remains unclear. Regardless, the result is that potential post-COVID sequelae may not be explicitly documented in registry data.

These coding practices and clinical framings may help explain the patterns observed in our data, where symptoms consistent with PASC appear under a range of diagnostic categories rather than being recognized as part of a coherent syndrome.

### The question of long-term immunodeficiency and immune dysregulation after COVID-19

The substantial increases we observed in respiratory infections in the periods following COVID-19 waves, particularly pediatric pneumonia, align with emerging evidence of post-COVID immune dysfunction. Immunological studies demonstrate persistent immune dysregulation lasting months after infection [43, 44]. Although more research is needed, the existing epidemiological evidence suggests that this has translated into increased infection rates in real-world populations [45]. Our temporal correlations—with increases in strep throat and pneumonia occurring after COVID-19 waves—are compatible with this immune dysfunction hypothesis [46–49]. The excesses in whooping cough and pediatric pneumonia corresponded to pertussis and mycoplasma pneumoniae epidemics, compatible with post-COVID immune dysfunction increasing population susceptibility. Notably, our results demonstrate the highest relative increases in infections in children and young people, age groups with the lowest coverage of COVID-19 vaccines. This opens for the question of whether increased vaccine uptake among younger individuals could have prevented some of this increase.

### Fatigue

Fatigue’s substantial deviations from pre-pandemic trends (started in 2020, worsened in 2022) is compatible with extensive evidence establishing fatigue as a primary post-acute COVID-19 sequela. A systematic review of 50 controlled studies (14 + million participants) found 58% increased fatigue risk following COVID-19 [50]. Studies report a lower risk of PASC in vaccinated individuals [51]; however, Norwegian data (57,000 participants) showed even triple-vaccinated individuals had 70% higher risk of prolonged fatigue after Omicron infection [17].

The coding patterns we observed likely reflect the absence of a specific PASC code in ICPC-2. Norwegian primary care physicians use A04 (weakness/tiredness general) when suspecting Myalgic Encephalomyelitis/Chronic Fatigue Syndrome (ME/CFS) [52], and the Norwegian Labour and Welfare Administration (NAV) found 182% increased risk of A04-related sick leave in the 12 weeks following COVID-19 sick leave compared to other illnesses [53]. Given that ME/CFS can develop in PASC patients [54], these increases are compatible with concerns about long-term functional consequences of a population experiencing repeated SARS-CoV-2 infections.

### Psychological complaints and stress reactions

The deviations from pre-pandemic trends for feeling depressed (started in 2021, worsened in 2023), acute stress reactions (started in 2020, worsened in 2023), and other psychological complaints (started in 2021, worsened in 2023) likely reflect multiple factors, including pandemic disruption, Norway's cost-of-living crisis, societal factors such as wars, the climate crisis, and both the potential neurobiological effects of SARS-CoV-2 and possible consequences of misclassification/psychologization of PASC. PASC is a deeply stigmatized disease, with over 80% of patients reporting stigma [55, 56]. Both stigmatization and psychologization of symptoms are associated with increased concerns about disclosure, reduced trust in medical care, elevated depression and anxiety, and greater loneliness, while also being linked to decreased life satisfaction and lower self-esteem [56]. However, studies also demonstrate increased risk of feeling depressed following COVID-19 [57, 58], with evidence pointing to neuroinflammation as a central mechanism in neuroimmunological PASC [59]. In line with this evidence, a certain level of increase in feeling depressed would be compatible with the consequences of widespread SARS-CoV-2 infections in Norway since 2022. However, a clear temporal association would not be expected. This is concordant with our data.

It is important to clarify that the code P03 (feeling depressed), which showed large excesses in 2024—both absolute (126,783) and relative (133%)—represents a basic disturbance in affect and mood, distinct to the disease of depression, which is classified under P76 (depression).

P29 (psychological symptom/complaint other) serves as a catch-all code for psychological issues without obvious sources. The Norwegian Directorate of Health’s ICPC-2 guide suggests P29 when searching for “slitenhet” (“*fatigue”*) [60], and it is used in practice for conditions including burnout [61], and suspected ME/CFS [52]. P02 (acute stress reaction) is used for stressful life events that impair social functioning [62], and is among the codes Norwegian primary care physicians employ when suspecting ME/CFS [52].

These coding practices may obscure post-COVID presentations, as diverse PASC symptoms may be coded under psychological categories despite physiological origins. NAV reports that (compared to people with sick leave for not COVID-19) people with COVID-19 sick leave were 35%, 25%, and 18% more likely to have subsequent depression, stress reaction, and P29-related sick leave, respectively [53]. This is compatible with the hypothesis that excess consultations for these conditions may be at least partly related to PASC.

### Cognitive impairment (memory disturbance and hyperkinetic disorder)

The deviations from pre-pandemic trends for memory disturbance (started in 2020, worsened in 2023) and hyperkinetic disorder (started in 2020, worsened in 2023) consultations are compatible with extensive evidence of cognitive impairment as a post-acute COVID-19 sequela. In accordance with this, a systematic review of 50 controlled studies (14 + million participants) found non-hospitalized COVID-19 patients had significantly increased risks of poor memory (RR 2.74) and poor concentration (RR 4.86) [50]. Norwegian studies showed 140% increased risk of poor memory and 100% increased risk of brain fog after Omicron infection in triple-vaccinated participants [17].

Both hyperkinetic disorder and memory disturbance demonstrated notable gender disparities in consultation patterns. Females recorded approximately 2–3 times higher relative excesses than males across all age groups for hyperkinetic disorder and twice the relative excesses among those aged 15–29 for memory disturbance. Studies have found the risk of PASC to be higher in females [63, 64], consistent with reports of distinct immunological processes of PASC in females and males [65]. Norwegian data also show in triple vaccinated individuals that the absolute risk of post-COVID-19 cognitive issues is higher in women than men [17], in line with the Global Burden of Disease Long Covid Collaborators who have reported a disproportionately high prevalence of PASC—including cognitive symptom clusters—in women [66]. Health seeking behaviour will also vary between the sexes and age groups [67], possibly affecting results. It is notable that the relative excess in memory problems is highest among children, while data for older adults in Norway, an age group where a very high percentage have received at least full primary series of three COVID-19 vaccines and most also one or more booster doses, have comparatively lower relative excesses. Differences in the perceived severity of cognitive symptoms may vary between age groups, for instance may young adults in educational training encounter more challenges than do older adults. Data suggest that Norwegian screen use, one complementary explanation to cognitive symptoms, plateaued or declined between 2021–2024 [68].

The neurological sequelae of COVID-19—concentration difficulties, impaired working memory, and mental flexibility problems [69, 70]—closely mirror ADHD symptoms. In addition, since 2021/2022, there has been a significant rise in Norwegian children with ADHD [71]. Although it is outside of the scope of this study, it is worth noting the concurrent rise in ADHD diagnoses, hyperkinetic disorder consultations, and widespread SARS-CoV-2 infections during this period. NAV researchers have directly attributed increased hyperkinetic disorder sick leave in working-age adults to PASC [72].

### Gastrointestinal complaints

The deviations from pre-pandemic trends for gastrointestinal complaints started in 2021. Abdominal pain is a symptom of acute COVID-19 [73]. However, in our data it was not significantly correlated with community spread of COVID-19 in the same quarter. Studies report gastrointestinal complaints such as nausea and abdominal pain as symptoms associated with post-acute sequelae of COVID-19 [74]. A systematic review found increased risks for long-term long-term abdominal pain (RR 1.44), nausea/vomiting (RR 1.47), and diarrhea (RR 1.31) in non-hospitalized COVID-19 patients [50].

In 2024, an internal NIPH investigation was launched to find the cause of the increase in primary healthcare consultations for gastritis, and found no increase in typical infectious gastroenteritis pathogens [23]. NAV findings report that (compared to other illnesses) people with COVID-19 sick leave were 176% more likely to have subsequent gastroenteritis-related sick leave [53]. Studies demonstrate altered gut microbiome [75] and gastrointestinal barrier disruption in fatigued PASC patients [76], suggesting that COVID-19 may disrupt pathways related to GI barrier function, leading to GI symptoms post-COVID-19. Thus, our findings are compatible with an expected increase in gastrointestinal complaints after periods of widespread SARS-CoV-2.

### Conjunctivitis

Conjunctivitis has been recognized as a symptom of acute COVD-19 [77]. The symptom became much more prevalent after the emergence of the omicron variant XBB.1.16 from mid 2023 [78]. This is compatible with our conjunctivitis findings, which showed mild deviations from the 2010–2019 baseline in 2023 and substantial deviations in 2024.

### Comparison with influenza patterns

COVID-19 is frequently compared to influenza, yet neither during the 2009–2010 H1N1 pandemic nor afterward did we observe increases in conditions now showing substantial excesses. This distinction suggests our observed patterns are specific to COVID-19 rather than reflecting general pandemic disruption or typical post-viral syndromes. The absence of similar H1N1-associated increases strengthens the hypothesis that current healthcare burden reflects unique SARS-CoV-2 characteristics and post-acute sequelae.

### Children and adolescents

Post-acute sequelae of COVID-19 are common in children and adolescents [79]. A Norwegian study found higher incidence of fatigue and brain fog 3–5 months after Omicron infection in 1500 children [17]. Our study observed substantial excesses in memory disturbance among children and adolescents, with girls and young women disproportionately affected.

Unlike other countries, Norway has not invested in information campaigns regarding prevention of COVID-19 or other measures to protect children such as school ventilation improvements [80]. Fewer than 10% of children aged 11–12 have received COVID-19 vaccines, with no booster recommendations [81–83], despite evidence that vaccination reduces pediatric PASC risk [51, 84, 85].

Our results are compatible with Norwegian children and young people experiencing significant excess morbidity as a result of the lack of vaccine recommendations.

### Comparison with previous study and international trends

Our results are consistent with our previous 2023 study [23], revealing the situation worsened in 2024 and also significantly affects children and adolescents—a troubling development given widespread repeated SARS-CoV-2 infections among Norwegian children since 2022.

These findings align with broader population health deterioration observed both nationally and internationally. In Norway, the period 2022–2024 with widespread SARS-CoV-2 transmission coincided with 13.0%, 8.4%, and 7.0% excess mortality respectively [86], representing an unprecedented level of excess mortality in Norway's modern public health history. This excess mortality, also affecting those under 65, is compatible with uncontrolled SARS-CoV-2 spread increasing deaths beyond acute SARS-CoV-2 infection [87].

In Europe, sick leave peaked in 2022 at + 0.56 percentage points above 2019 levels, however, sick leave levels remained elevated at + 0.32 percentage points in 2024, with 68% of countries showing higher rates than in 2019 [88]. After accounting for pre-pandemic trends (2010–2019), life expectancy in 2024 was numerically lower than forecast for 88% of EU countries, with an average deficit of 0.27 years [89]. Given that EU life expectancy increased by an average of 0.18 years annually from 2010–2019, this 0.27-year deficit means EU life expectancy is now approximately 1.5 years behind the trajectory established before the pandemic.

There are also reports of larger shifts: In Spain, self-reported chronic health problems increased from stable 30% (2012–2019) to 50% by late 2024 [90–93]. In the United States, disability numbers remained steady at 29–31 million until 2019, then rose continuously to nearly 35 million by end-2024 [94].

These reports suggest our results reflect genuine shifts in population health rather than measurement artifacts or Norway-specific phenomena.

### Alternative hypotheses than COVID-19

A more detailed discussion of alternative hypotheses than COVID-19 is available in Additional File 7.

Before considering alternative hypotheses, it is important to address the apparent contradiction between declining diagnosis codes for acute COVID-19 and the hypothesis of ongoing SARS-CoV-2 impact. The decline in consultations with direct COVID-19 codes (R991 + R992) from 2023 onwards does not represent a true magnitude of reduced transmission but rather changes in testing policy. From 2023, for the majority of the population, the Norwegian Institute of Public Health recommended against testing for COVID-19 when symptomatic [11]. This has led to few being tested for COVID-19, including patients presenting with symptoms of respiratory infection at GP consultations and emergency room visits, resulting in substantial underreporting despite continued widespread SARS-CoV-2 circulation.

The composition of consultations for respiratory infections has shifted substantially over time. In the early pandemic period (2020–2022), acute COVID-19 codes (R991 + R992) accounted for 70.1%, 53.8%, and 67.5% of excess consultations in 2020, 2021, and 2022 respectively. By 2024, R991 + R992 accounted for 5.1% of excess consultations, yet respiratory infections remained substantially elevated with striking increases in whooping cough (343% relative excess), pneumonia in children aged 5–14 (1,066% relative excess), and cough in the same age group (257% relative excess). This temporal shift suggests the respiratory infection burden now reflects a combination of miscategorized acute COVID-19 cases and increased susceptibility to other respiratory pathogens, consistent with documented post-acute sequelae features including immune dysregulation and increased infection susceptibility [45]. However, temporal correlations between respiratory infection increases and COVID-19 community spread waves, combined with persistence two years after mitigations ended in 2022, suggest factors beyond simple rebound effects contribute to observed patterns.

**Fig. 5:**
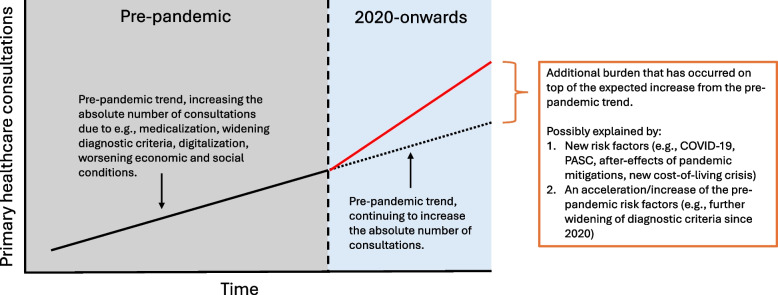
Conceptual framework for distinguishing pre-pandemic trends from post-2020 increases in primary healthcare consultations

When considering hypotheses other than COVID-19, they must account for the fact that 2024 consultation rates exceeded pre-pandemic trend projections. Pre-existing factors cannot explain these increases unless underlying trends accelerated after 2020 (Fig. 5).

A previous study noted an increase in ADHD-medication from 2010–2020 across Scandinavia [95], consistent with observed increases in primary healthcare consultations for hyperkinetic disorder from 2010–2019. Some researchers have suggested that this may relate to increased medicalization [96] or widening diagnostic criteria [97]. Crucially, however, our study observed an even larger increase from 2020 onwards that rapidly outpaced pre-pandemic trends, also observed in Denmark [97]. This suggests either a new risk factor has occurred (e.g., COVID-19) or pre-pandemic causes have accelerated post-2020, or both (Fig. 5).

Regarding psychological complaints, an important consideration is whether our findings reflect COVID-19 impacts versus broader national or global mental health trends [98]. The magnitude and sustained elevation from 2022–2024 suggest an acute acceleration beyond historical trends. A similar increase has been observed for medically certified sick leave [72]. According to the Norwegian Labour and Welfare Administration, the post-2021 increase in medically certified sick leave for psychological complaints resulted from both longer leave durations and more individuals taking such leave [72]. Sick leave for psychological issues increased across the entire Norwegian population, indicating that there are factors influencing the whole population, not just vulnerable groups, at play [72]. Ultimately, the authors found no support for the hypotheses that the increase in psychological issues is caused by social media, changes in sick leave incentives, internet/screen-time usage, increased work pressure, understaffing, or increase in home office usage [72].

While healthcare digitalization may contribute to increased consultation rates by improving access, it cannot fully explain observed patterns. If digitalization were the primary driver, we would expect relatively uniform increases across conditions amenable to telemedicine. Instead, psychological symptoms (80% + relative excess), hyperkinetic disorder (116%), memory disturbance (63%), and fatigue (70%) far exceeded the relative excesses in other telemedicine-suitable conditions such as skin rashes (30%). The mean annual number of consultations per general practitioner also returned to pre-pandemic levels by 2023 [23], indicating that by 2023 e-consultations replaced physical consultations without additional healthcare seeking behavior.

Delayed healthcare, pent-up demand, general pandemic disruption, and economic stress could theoretically contribute to increased primary healthcare utilization. However, several observations argue against these as fully explaining the observed patterns. Due to effective mitigation strategies, Norway’s healthcare was not overwhelmed during the acute phase of the pandemic [99]. Pent-up demand typically manifests as a temporary spike followed by return to baseline, whereas our data show sustained elevation through 2024 with consultation rates remaining 7.1% above expected levels. The diagnostic specificity of increases—concentrated in conditions compatible with PASC such as fatigue, memory disturbance, and respiratory symptoms—is inconsistent with general patterns of delayed routine or preventive care. Temporal correlations between increases and SARS-CoV-2 circulation patterns, combined with persistence through 2024 despite economic recovery [100], suggest factors beyond pandemic disruption and economic stress are at play.

### Limitations

The fundamental limitation of this study is that it is a study based on aggregate data analyzing temporal changes. While these condition-specific findings are suggestive, the aggregate and ecological nature necessitates caution in causal attribution. The design cannot control for other pandemic-associated factors that may have increased healthcare demand, such as feeling depressed from preventative measures, activity loss, or bereavement.

Our estimates include data on digital consultations conducted during out-of-office hours. However, there may be coding inaccuracies in these data, as the higher reimbursement rate for digital out-of-office hours consultations compared to administrative patient contact creates a potential financial incentive for miscoding [101]. This adds some uncertainty as to the estimates for excess consultations. However, the data show a clear and substantial increase also when not including these consultations, with a total of 17,170,953 consultations, corresponding to an excess of 836,033 consultations, or a 5.1% relative excess compared to expected levels.

The study cannot capture full SARS-CoV-2 spread due to limited community transmission data, though our proxy measure showed positive temporal correlations across conditions. NorSySS captures only 102 of 710 available ICPC-2 codes, focusing on infectious diseases and some post-acute syndromes. Since PASC affects multiple organ systems, our findings may represent a subset of broader healthcare changes. However, our ten highest absolute excess conditions accounted for 112% of the total observed excess of 1,185,231, indicating we captured the most significant increases. The total is lower because it represents the net balance across all conditions—while some conditions had substantial increases, others had fewer consultations than expected, partially offsetting the overall excess. For unmeasured ICPC-2 codes to account for substantial absolute excesses, they would require corresponding deficits in measured codes of equivalent magnitude. We observed no such large deficits within NorSySS' 102 codes, suggesting that simultaneous large increases and offsetting decreases are not characteristic of this dataset. Furthermore, our previous study examining all ICPC-2 codes for medically certified sick leave in 2023 found substantially similar results, providing independent validation across complete diagnostic coverage [23]. However, this does not hold true for relative excesses, and hence a notable limitation is that there may be large relative excesses in the 608 unmeasured codes, especially for less common ICPC-2 codes.

Our data cannot distinguish between acute COVID-19 and PASC consultations. Observed increases may reflect early post-infection symptoms (some resolving, others evolving into chronic PASC), established PASC cases (3 + months post-infection), or combinations thereof. Given the lack of definitive PASC treatments [16], patients with persistent symptoms may discontinue seeking healthcare after initial contact, meaning our study may capture initial healthcare engagement but miss subsequent months or years of ongoing illness. This suggests our findings likely underestimate the true health impact of SARS-CoV-2 infections, as population-level health burdens extend beyond what is captured in primary healthcare utilization data.

### Strengths

This study utilizes national-level NorSySS registry data ensuring near-complete coverage of Norwegian primary healthcare consultations, including both GP and out-of-hours visits. Age and sex stratification revealed differential impacts among children, adolescents, and adults. The study expands upon our previous work, establishing that concerning 2023 trends persisted and worsened in 2024.

Incorporation of a COVID-19 community spread proxy enabled exploration of temporal correlations. The temporal correlation analysis provides additional validation of our findings. Conditions with the strongest biological evidence for COVID causation showed the strongest temporal correlations with COVID-19 community spread. Respiratory infections like strep throat (*r* = 0.64), pneumonia (*r* = 0.46), and conjunctivitis (*r* = 0.65)—which have clear mechanistic links to post-COVID immune dysfunction—demonstrated the strongest correlations in the prior quarter. Similarly, fatigue (*r* = 0.58) and memory disturbance (*r* = 0.50), well-established post-acute COVID-19 sequelae, showed notable temporal associations. In contrast, conditions with weaker biological plausibility for COVID causation generally showed weaker correlations, supporting our interpretation that some of the observed increases are compatible with genuine COVID-19 health impacts.

## Conclusions

Substantial increases were observed in primary healthcare consultations in 2024 compared to pre-pandemic levels. Many of the conditions with the greatest excess are associated with post-acute COVID-19 sequelae, supporting a notion that the lack of a specific diagnosis for PASC in the ICPC coding system used by GPs in Norway may have contributed to obscuring the prevalence of this condition. The findings are compatible with the hypothesis that the ongoing population-level health impacts are associated with repeated SARS-CoV-2 infections, particularly among women, children, adolescents, and young adults. Evidence from this study is compatible with children in Norway experiencing excess morbidity related to a lack of protection from SARS-CoV-2 vaccines. Given that Norway's COVID-19 strategy and risk assessments do not address PASC, this information provides critical evidence for understanding the population health consequences of policies that encourage repeated SARS-CoV-2 infections. These healthcare utilization patterns, together with our previous findings of increased sick leave and concurrent observations of excess mortality during 2022–2024, suggest that inaction in the face of these signals may institutionalize chronic illness within the population, with far-reaching consequences for workforce participation, healthcare capacity, and national economic stability. More research is needed to establish clearer associations between symptoms and long-term effects of SARS-CoV-2 infections, and their consequences for healthcare utilization and society as a whole.

## Supplementary Information

Additional file 1. Estimates for 101 ICPC-2 code combinations and all sex and age combinations.

Additional file 2. Seasonality analysis of COVID-19 in Norway.

Additional file 3. Different measures of COVID-19 community spread between 2020-W09 and 2024-W52.

Additional file 4. Ratio of 2024 incidence against 2010–2019 modelled baseline and correlation within COVID-19 community spread for ICPC-2 codes A*, B*, D*, F*, H*, N*, P*, R*, S*, U*, X*, Y*.

Additional file 5. Trend graphs and residual diagnostic plots for the results in Additional File 1.

Additional file 6. Detailed results supplement.

Additional file 7. Detailed alternate theories than COVID-19 supplement.

## Data Availability

The data that support the findings of this study are available from the Norwegian Syndromic Surveillance System, which were used under license for the current study, and so are not publicly available. Applications for data may be made to the Norwegian Syndromic Surveillance System.

## Abbreviations

COVID-19: Coronavirus disease 2019
FDR: False discovery rate
ICPC-2: International Classification of Primary Care version 2
KPR: Municipal Patient and User Register; Norwegian: Kommunalt pasient- og brukerregister
KUHR: Control and Payment of Health Reimbursements; Norwegian: Kontroll og utbetaling av helserefusjoner
ME/CFS: Myalgic Encephalomyelitis/Chronic Fatigue Syndrome
NIPH: Norwegian Institute of Public Health; Norwegian: Folkehelseinstituttet
NorSySS: Norwegian Syndromic Surveillance System; Norwegian: Det norske syndromiske overvåkingssystemet
PCR: Polymerase chain reaction
PI: Prediction interval
RNA: Ribonucleic acid
SARS-CoV-2: Severe acute respiratory syndrome coronavirus 2
