# Supplementary material for: Excess primary healthcare consultations in Norway in 2024 compared to pre-COVID-19-pandemic baseline trends
Source: Arch Public Health. 2026 Jan 2;84:26. doi: 10.1186/s13690-025-01817-8 (PMC12866491; doi:10.1186/s13690-025-01817-8)
Supplement: Supplementary file 2 — Additional file 2. Seasonality analysis of COVID-19 in Norway. [file 13690_2025_1817_MOESM2_ESM.docx]

Table S2. Description of COVID-19 community spread for each month between 2020-W09 and 2024-W52.

| Month | 2020 | 2021 | 2022 | 2023 | 2024 |
| --- | --- | --- | --- | --- | --- |
| Jan |  | Low | Increasing | Decreasing | Decreasing |
| Feb |  | Low | Increasing | Trough | Decreasing |
| Mar | Low | Low | Peak | Increasing | Trough |
| Apr | Low peak | Low peak | Decreasing | Peak | Trough |
| May | Low | Low | Trough | Decreasing | Trough |
| Jun | Low | Low | Peak | Decreasing | Increasing |
| Jul | Low | Low | Decreasing | Trough | Peak |
| Aug | Low | Increasing | Decreasing | Increasing | Peak |
| Sep | Low | Peak | Trough | Peak | Decreasing |
| Oct | Low | Increasing | Increasing | Increasing | Decreasing |
| Nov | Low peak | Increasing | Increasing | Peak | Decreasing |
| Dec | Low | Peak | Peak | Decreasing | Trough |
