# Supplementary figures and images for: Excess primary healthcare consultations in Norway in 2024 compared to pre-COVID-19-pandemic baseline trends
Source: Arch Public Health. 2026 Jan 2;84:26. doi: 10.1186/s13690-025-01817-8 (PMC12866491; doi:10.1186/s13690-025-01817-8)

Figure S2. Different measures of COVID-19 community spread between 2020-W09 and 2024-W52.

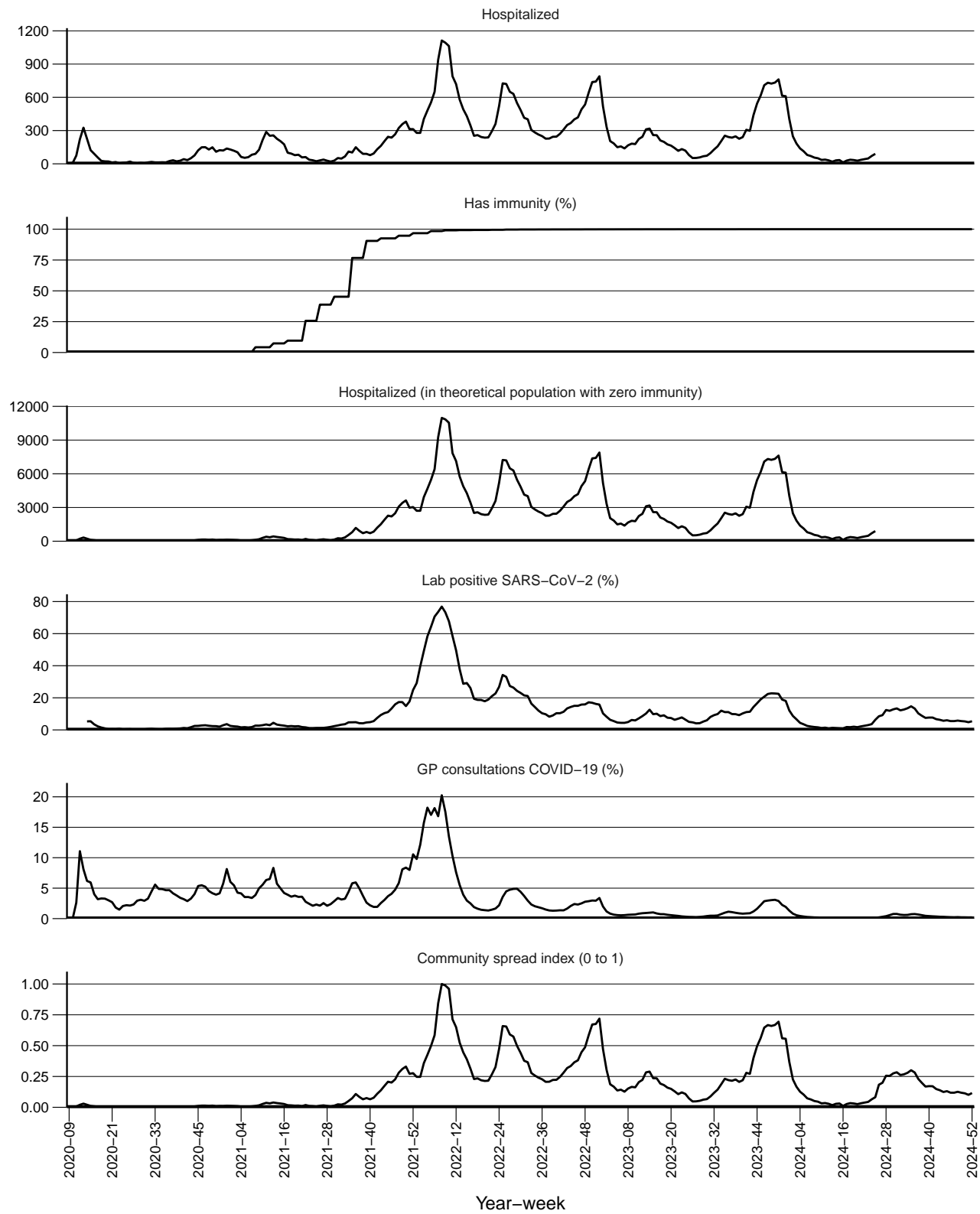

Supplement: Supplementary file 3 — Additional file 3. Different measures of COVID-19 community spread between 2020-W09 and 2024-W52. [file 13690_2025_1817_MOESM3_ESM.pdf]
