# Supplementary material for: Excess primary healthcare consultations in Norway in 2024 compared to pre-COVID-19-pandemic baseline trends
Source: Arch Public Health. 2026 Jan 2;84:26. doi: 10.1186/s13690-025-01817-8 (PMC12866491; doi:10.1186/s13690-025-01817-8)
Supplement: Supplementary file 4 — Additional file 4. Ratio of 2024 incidence against 2010–2019 modelled baseline and correlation within COVID-19 community spread for ICPC-2 codes A*, B*, D*, F*, H*, N*, P*, R*, S*, U*, X*, Y*. [file 13690_2025_1817_MOESM4_ESM.pdf]

Figure S3. Ratio of 2024 incidence against 2010−2019 baseline and correlation within COVID−19 community spread for ICPC−2 codes A*, B*, D*, F*, H*, N*, P*.

	All sexes						Female						Male						Correlation with COVID−19 community spread			
	All ages	5−14 years old	15−19 years old	20−29 years old	30−64 years old	65+ years old	All ages	5−14 years old	15−19 years old	20−29 years old	30−64 years old	65+ years old	All ages	5−14 years old	15−19 years old	20−29 years old	30−64 years old	65+ years old	Same quarter	Previous quarter	Two quarters ago	
A02 Chills																			0.48			A
A03 Fever	1.7	1.9		2.3	2.1	1.3	1.7			2.2	2.1	1.3	1.7	1.9		2.4	2.0	1.4	0.51	0.63		A
A04 Weakness/tiredness general	1.7	1.5	1.3	1.5	1.8	1.6	1.7	1.5	1.3	1.6	1.8	1.6	1.7	1.4		1.5	1.8	1.7	0.45	0.58		A
A05 Feeling ill	0.7	0.4	0.7	0.6	0.6	0.8	0.7	0.4	0.7	0.6	0.7	0.8	0.6	0.3		0.6	0.6	0.8	0.61	0.57		A
A27 Fear of other diseases NOS	2.0	1.9	2.1	1.9	1.8	2.7	2.0	2.0	1.9	2.1	1.8	2.5	2.0	1.9	2.2	1.7	1.8	3.2	−0.60	−0.62	−0.57	A
A71 Measles			0.2			0.3			0.0									0.0				A
A72 Chickenpox																			0.49	0.63	0.60	A
A74 Rubella			0.0			0.0		3.0	0.0			0.0	0.3		0.0	0.0		0.0				A
A75 Infectious mononucleosis			0.8		1.9				0.8		1.8				0.7				0.49	0.51	0.44	A
A76 Viral exanthem other	2.0	2.0		2.2	2.2		2.1	2.0		2.1	3.4		1.8	2.0					0.59			A
A77 Viral disease other/NOS	2.0			4.1	3.1	2.2	2.4			4.5	3.9	3.8	1.6			3.8	2.4					A
A78 Infectious disease other/NOS	1.8	4.1	1.9	2.2	1.9	1.3	2.0	4.4	1.9	2.3	2.1	1.4	1.6	3.9	2.0	2.1	1.7				0.53	A
B02 Lymph gland(s) enlarged/painful	1.5	1.5	1.7	1.5	1.5	1.6	1.6		1.7	1.6	1.6	2.0	1.5									B
B25 Fear of AIDS/HIV	2.0	0.5			2.1			0.0	0.0				2.0				2.1			0.56	0.62	B
B70 Lymphadenitis acute	1.3	1.4					1.3							1.5								B
B71 lymphadenitis non−specific	1.4		1.7				1.5															B
D01 Abdominal pain/cramps general	1.3			1.4	1.4	1.3	1.3	1.2		1.5	1.4	1.4	1.2				1.3	1.3		0.49		D
D02 Abdominal pain epigastric	1.1				1.1		1.1			1.2	1.2											D
D06 Abdominal pain localized other	1.0						1.0		1.2	1.1												D
D08 Flatulence/gas/belching	1.3				1.4		1.2				1.4		1.4				1.5	1.6				D
D09 Nausea	1.5	1.5	0.8	1.6	2.0	1.7	1.6	1.5		1.8	2.0	1.7	1.4	1.4	0.7		2.0	2.0		0.51		D
D10 Vomiting	1.3	1.2	0.6	1.5	1.9	1.6	1.4		0.6	1.6	2.0	1.7		1.2	0.6		1.8	1.5	0.54	0.63		D
D11 Diarrhea	1.4	1.3	0.7	1.4	1.7	1.4	1.5		0.7	1.5	1.8	1.4	1.3	1.3	0.6	1.3	1.5	1.4	0.49	0.52	0.49	D
D11+D70+D73 Gastroenteritis	1.2	1.2	0.5	1.4	1.5	1.3	1.3		0.5	1.5	1.6	1.3	1.1	1.2	0.5	1.2	1.4	1.3	0.51	0.65	0.47	D
D14 Hematemesis/vomiting blood	1.2				1.2	1.2					1.3		1.2			1.4		1.3		0.54		D
D18 Change feces/bowel movements	1.4	1.3		1.2	1.6	1.4	1.4			1.2	1.6		1.5	1.5	1.5		1.5			0.50		D
D25 Abdominal distension	1.4						1.4															D
D29 Digestive symptom/complaint other	1.6	1.3		1.6	1.6	1.8	1.8	1.4		1.8	1.9	2.0	1.4				1.4	1.6				D
D70 Gastrointestinal infection	1.4	1.5	0.6	1.6	1.6	1.2	1.4	1.6	0.7	1.6	1.6	1.4	1.3	1.4	0.6	1.7	1.6		0.51	0.60	0.53	D
D73 Gastroenteritis presumed infection			0.4	1.3	1.4				0.4	1.4	1.5				0.4				0.48	0.68		D
D87 Stomach function disorder	1.2		0.7		1.2		1.2			1.6	1.3											D
D99 Disease digestive system, other	1.3	1.5	1.5	1.7	1.4		1.4	1.5	1.6	2.0	1.4		1.3	1.5			1.4					D
F70 Conjunctivitis infectious	1.6	2.5		1.8	1.6	1.2	1.7	2.7		2.0	1.8	1.2	1.4	2.3		1.6	1.4	1.2		0.65	0.54	F
F73 Eye infection/inflammation other		2.2						2.3											0.51	0.65	0.57	F
H01 Ear pain/earache	1.9	2.1	1.4	1.9	2.0	1.8	2.0	2.1	1.4	2.0	2.0	2.2	1.8	2.1			2.0			0.63		H
H04 Ear discharge	3.4	4.6	6.6	2.7	5.0		3.0						4.0			43.0	163.0			0.63	0.49	H
H13 Plugged feeling ear	1.2						1.3													0.57	0.57	H
H29 Ear symptom/complaint other	1.3	1.5			1.3		1.3	1.6		1.3	1.3		1.3	1.4						0.62	0.56	H
H71 Acute otitis media/myringitis	1.5	1.7	1.2	1.5	1.5	1.2	1.5	1.8		1.5	1.5	1.3	1.5	1.7		1.4	1.4			0.66		H
H77 Perforation ear drum			0.7										0.9		0.6					0.72	0.60	H
N16 Disturbance of smell/taste	0.8					0.7	0.7	3.4			0.8	0.6							0.57			N
P02 Acute stress reaction	1.8	1.9		1.8	1.8	2.1	1.8	2.0		1.8	1.8	2.1	1.7	1.7		1.6	1.8	2.0				P
P03 Feeling depressed	2.3	1.6		2.1	2.7	2.1	2.3	1.6		2.2	2.7	2.1	2.3	1.5		1.9	2.8	2.2				P
P20 Memory disturbance	1.6	2.7	2.1	2.3	1.7	1.3	1.7	3.2	2.5	2.7	1.8	1.3	1.6	2.5	1.7	1.9	1.7	1.3		0.50	0.45	P
P29 Psychological symptom/complaint other	1.9	1.5	1.2	1.8	2.0	1.8	1.9	1.6	1.2	1.8	2.0	1.8	1.8			1.6	1.9	1.8				P
P81 Hyperkinetic disorder	2.2	2.0	2.1	2.2	2.2	2.0	2.6	2.3	2.7	2.9	2.5	2.7	1.8	1.8	1.7	1.6	2.0	1.6				P

Ratio of 2024 incidence against 2010−2019 modelled baseline: Deficit (<0.6) | Deficit (0.6 to 0.9) | Not significant (FDR) | Excess (1.1 to 1.5) | Excess (>1.5)

Numbers in left three panels are at the annual level.
Right panel shows significant (p<0.05) correlation (r) with COVID−19 community spread, and was performed for all ages and sexes combined at quarterly level.

Figure S4. Ratio of 2024 incidence against 2010−2019 baseline and correlation within COVID−19 community spread for ICPC−2 codes R*, S*, U*, X*, Y*.

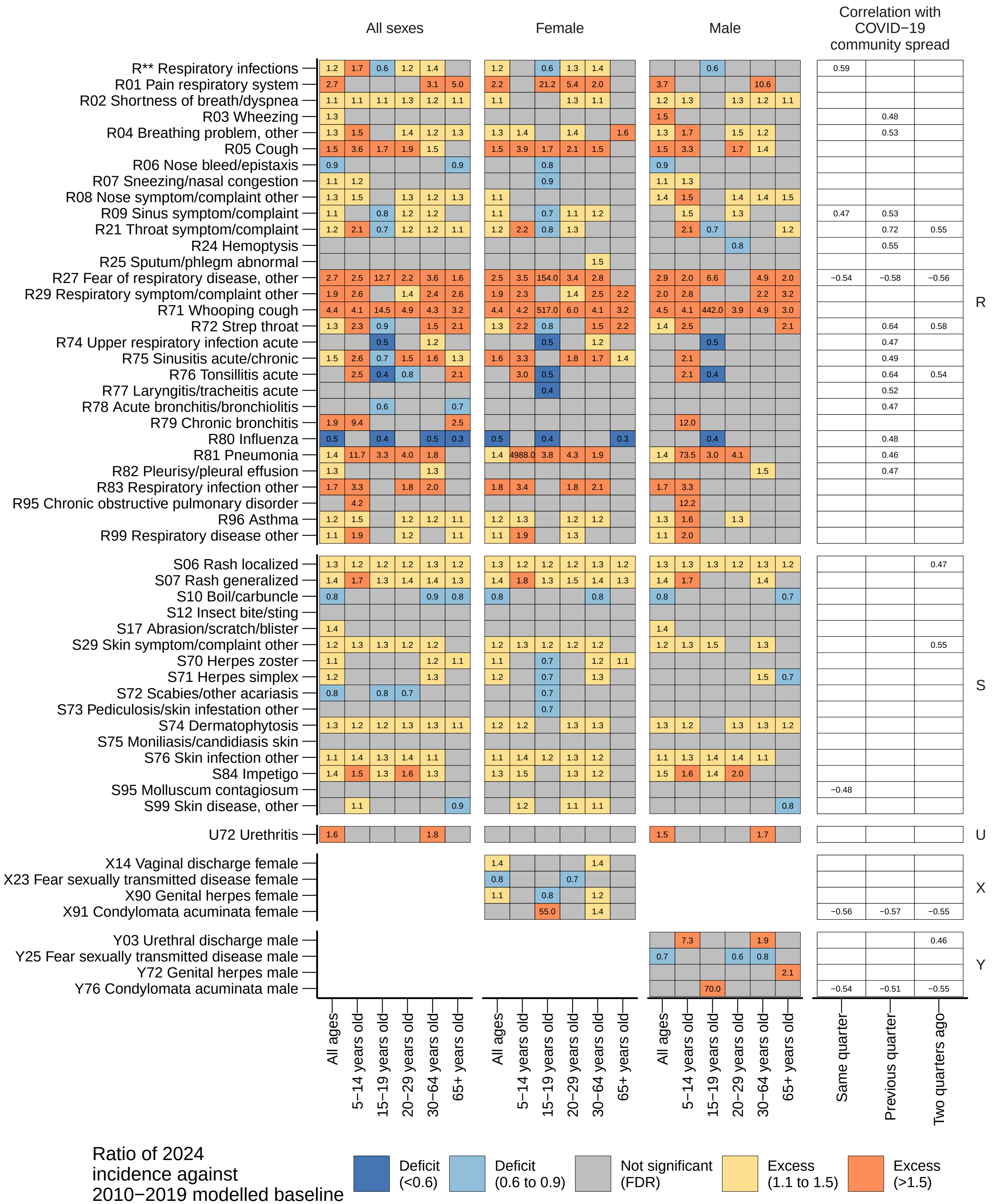

Numbers in left three panels are at the annual level.
Right panel shows significant ($p<0.05$) correlation (r) with COVID−19 community spread, and was performed for all ages and sexes combined at quarterly level.
