# Supplementary material for: Excess primary healthcare consultations in Norway in 2024 compared to pre-COVID-19-pandemic baseline trends
Source: Arch Public Health. 2026 Jan 2;84:26. doi: 10.1186/s13690-025-01817-8 (PMC12866491; doi:10.1186/s13690-025-01817-8)
Supplement: Supplementary file 6 — Additional file 6. Detailed results supplement. [file 13690_2025_1817_MOESM6_ESM.docx]

**Additional File 6.** Detailed results supplement.

**Respiratory infections**

R** is a composite code, consisting of R01, R02, R03, R04, R05, R07, R08, R09, R21, R24, R25, R27, R29, R33, R71, R72, R74, R75, R76, R77, R78, R79, R80, R81, R82, R83, R99, R991, and R992 (Additional file 1).

In 2024, R** (respiratory infections) exceeded the modelled baseline by 325,726 consultations (95% PI: 144,288 to 506,189)—a relative excess of 20% (95% PI: 8% to 36%) (Figures 3 and S4 and Table S1). While there was variation in relative excess between age groups, the overlapping prediction intervals (ranging 10-80%) indicate these differences were not statistically significant, with the exception of 15-19 year-olds who had 37% fewer consultations than expected. There was no noticeable variation in relative excess by sex (Additional file 5, Table S78). Deviation from expected pre-pandemic trends began in 2020, with a larger excess occurring in 2022, followed by lower but still elevated excess in 2023 and 2024 (Additional file 5, Figure S155). Temporal correlations with COVID-19 community spread were 0.59 (95% CI: 0.20 to 0.82) in the same quarter, 0.20 (95% CI: -0.26 to 0.59) in the prior quarter, and -0.19 (95% CI: -0.58 to 0.27) two quarters prior.

Within the composite code, R05 (cough) exceeded the modelled baseline by 91,342 consultations (95% PI: 72,401 to 110,361)—a relative excess of 53% (95% PI: 38% to 73%) (Figures 3 and S4 and Table S1). Relative excess varied considerably across age groups. Children aged 5–14 showed the highest relative excess at 257%. Age groups 15–19, 20–29, and 30–64 had overlapping prediction intervals with central estimates ranging from 50–90%. There was no noticeable variation in relative excess by sex (Figure 3 and Additional file 5, Table S53). Temporal correlations with COVID-19 community spread were 0.35 (95% CI: -0.11 to 0.69) in the same quarter, 0.43 (95% CI: -0.02 to 0.73) in the prior quarter, and 0.14 (95% CI: -0.33 to 0.55) two quarters prior.

Within the composite code, R83 (respiratory infection other) exceeded the modelled baseline by 78,382 consultations (95% PI: 41,318 to 112,734)—a relative excess of 73% (95% PI: 29% to 153%) (Figures 3 and S4 and Table S1). Relative excess varied considerably across age groups. Children aged 5–14 showed the highest relative excess at 226%, while 15–19 year olds and 65+ year olds were not significantly higher than the modelled baseline. There was no noticeable variation in relative excess by sex (Figure 3 and Additional file 5, Table S74). Temporal correlations with COVID-19 community spread were 0.24 (95% CI: -0.22 to 0.62) in the same quarter, 0.39 (95% CI: -0.07 to 0.71) in the prior quarter, and 0.11 (95% CI: -0.35 to 0.53) two quarters prior.

Within the composite code, R81 (pneumonia) exceeded the modelled baseline by 38,370 consultations (95% PI: 19,793 to 56,763)—a relative excess of 41% (95% PI: 18% to 76%) (Figures 3 and S4 and Table S1). Relative excess varied across age groups. 65+ year olds were not significantly higher than the modelled baseline, while 5–14 year olds had a relative excess of 1066% (95% PI: 135% to 534,800%). There was no noticeable variation in relative excess by sex (Figure 3 and Additional file 5, Table S72). Temporal correlations with COVID-19 community spread were 0.26 (95% CI: -0.21 to 0.63) in the same quarter, 0.46 (95% CI: 0.03 to 0.75) in the prior quarter, and 0.26 (95% CI: -0.21 to 0.63) two quarters prior.

Within the composite code, R72 (strep throat) exceeded the modelled baseline by 11,935 consultations (95% PI: 7,171 to 16,692)—a relative excess of 41% (95% PI: 18% to 76%) (Figures 3 and S4 and Table S1). Relative excess varied across age groups. 15–19 were significantly lower than the modelled baseline, 20–29 year olds were not significantly higher than the modelled baseline, while other age groups had overlapping prediction intervals. No significant differences were observed between males and females for relative excess (Figure 3 and Additional file 5, Table S64). Temporal correlations with COVID-19 community spread were 0.35 (95% CI: -0.11 to 0.69) in the same quarter, 0.64 (95% CI: 0.27 to 0.84) in the prior quarter, and 0.58 (95% CI: 0.19 to 0.82) two quarters prior.

Within the composite code, R71 (whooping cough/pertussis) exceeded the modelled baseline by 8,074 consultations (95% PI: 7,409 to 8,705)—a relative excess of 343% (95% PI: 246% to 506%) (Figures 3 and S4). Relative excess did not vary across age groups. No significant differences were observed between males and females for relative excess (Figure 3 and Additional file 5, Table S64). Temporal correlations with COVID-19 community spread were -0.07 (95% CI: -0.49 to 0.39) in the same quarter, -0.01 (95% CI: -0.45 to 0.44) in the prior quarter, and 0.04 (95% CI: -0.41 to 0.47) two quarters prior.

**Weakness/tiredness general (Fatigue) (A04)**

In 2024, A04 (weakness/tiredness general) exceeded the modelled baseline by 205,381 consultations (95% PI: 186,161 to 224,576)—a relative excess of 70% (95% PI: 60% to 82%) (Figures 2 and S3 and Table S1). There was no noticeable variation in relative excess by age or sex (Additional file 5, Table S5).

Temporal correlations with COVID-19 community spread were 0.45 (95% CI: 0.01 to 0.75) in the same quarter, 0.58 (95% CI: 0.19 to 0.82) in the prior quarter, and 0.44 (95% CI: -0.00 to 0.74) two quarters prior. Deviation from expected pre-pandemic trends began in 2021, but worsened dramatically from 2022 to 2024 (Additional file 5, Figure S9).

**Psychological symptom/complaint other (P29)**

In 2024, P29 (psychological symptom/complaint other) exceeded the modelled baseline by 188,978 consultations (95% PI: 176,676 to 201,509)—a relative excess of 87% (95% PI: 77% to 99%) (Figures 2 and S3 and Table S1). Relative excess was lower in 5–14 year olds (46%) and 15–19 year olds (15%) than older age groups (central estimates between 78–95%). There was no noticeable variation in relative excess by sex (Additional file 5, Table S47).

Temporal correlations with COVID-19 community spread were 0.24 (95% CI: -0.22 to 0.62) in the same quarter, 0.40 (95% CI: -0.06 to 0.71) in the prior quarter, and 0.34 (95% CI: -0.12 to 0.68) two quarters prior. Deviation from expected pre-pandemic trends began in 2020, but worsened dramatically from 2023 to 2024 (Additional file 5, Figure S93).

**Acute stress reaction (P02)**

In 2024, P02 (acute stress reaction) exceeded the modelled baseline by 182,079 consultations (95% PI: 165,064 to 198,907)—a relative excess of 76% (95% PI: 64% to 89%) (Figures 2 and S3 and Table S1). Relative excess was not significantly higher than the modelled baseline in 5–14 year olds (46%), while other age groups had overlapping prediction intervals. There was no noticeable variation in relative excess by sex (Additional file 5, Table S44).

Temporal correlations with COVID-19 community spread were 0.25 (95% CI: -0.22 to 0.62) in the same quarter, 0.39 (95% CI: -0.07 to 0.71) in the prior quarter, and 0.34 (95% CI: -0.12 to 0.68) two quarters prior. Deviation from expected pre-pandemic trends began in 2020, but worsened dramatically from 2023 to 2024 (Additional file 5, Figure S87).

**Feeling depressed (P03)**

In 2024, P03 (feeling depressed) exceeded the modelled baseline by 126,783 consultations (95% PI: 109,110 to 144,499)—a relative excess of 133% (95% PI: 97% to 187%) (Figures 2 and S3 and Table S1). Relative excess was not significantly higher than the modelled baseline in 15–19 year olds, while 20–29 year olds, 30–64 year olds, and 65+ year olds had overlapping prediction intervals in the 110–170% range. There was no noticeable variation in relative excess by sex (Additional file 5, Table S45).

Temporal correlations with COVID-19 community spread were 0.28 (95% CI: -0.19 to 0.64) in the same quarter, 0.39 (95% CI: -0.06 to 0.71) in the prior quarter, and 0.33 (95% CI: -0.13 to 0.68) two quarters prior. Deviation from expected pre-pandemic trends began in 2021, but worsened dramatically from 2023 to 2024 (Additional file 5, Figure S89).

**Hyperkinetic disorder (P81)**

In 2024, P81 (hyperkinetic disorder) exceeded the modelled baseline by 112,763 consultations (95% PI: 108,974 to 116,531)—a relative excess of 116% (95% PI: 108% to 125%) (Figures 2 and S3 and Table S1). Females had double to triple the relative excess of males, depending on the age group (Additional file 5, Table S48).

Temporal correlations with COVID-19 community spread were 0.37 (95% CI: -0.09 to 0.70) in the same quarter, 0.40 (95% CI: -0.05 to 0.72) in the prior quarter, and 0.42 (95% CI: -0.03 to 0.73) two quarters prior. Deviation from expected pre-pandemic trends began in 2020, but worsened dramatically from 2023 onwards (Additional file 5, Figure S95).

**Abdominal pain/cramps general (D01)**

In 2024, D01 (abdominal pain/cramps general) exceeded the modelled baseline by 84,544 consultations (95% PI: 69,965 to 99,041)—a relative excess of 29% (95% PI: 23% to 36%) (Figures 2 and S3 and Table S1). Relative excess was not significantly higher than the modelled baseline in 15–19 year olds, while 20–29 year olds, 30–64 year olds, and 65+ year olds had overlapping prediction intervals in the 35–39% range. There was no noticeable variation in relative excess by sex (Additional file 5, Table S19).

Temporal correlations with COVID-19 community spread were 0.36 (95% CI: -0.10 to 0.69) in the same quarter, 0.49 (95% CI: 0.07 to 0.77) in the prior quarter, and 0.33 (95% CI: -0.13 to 0.68) two quarters prior. Deviation from expected pre-pandemic trends began in 2021 (Additional file 5, Figure S37).

**Memory disturbance (P20)**

In 2024, P20 (memory disturbance) exceeded the modelled baseline by 39,177 consultations (95% PI: 36,988 to 41,378)—a relative excess of 63% (95% PI: 58% to 69%) (Figures 2 and S3 and Table S1). Relative excess varied significantly with age, with 5–14 year olds (174%) having the largest relative excess, followed by 20–29 year olds (129%), 15–19 year olds (110%), 30–64 year olds (74%), and 65+ year olds (32%). Females had approximately double the excess of males in the age groups 5–14 years old, 15–19 years old, and 20–29 years old (Additional file 5, Table S46).

Temporal correlations with COVID-19 community spread were 0.39 (95% CI: -0.06 to 0.71) in the same quarter, 0.50 (95% CI: 0.08 to 0.77) in the prior quarter, and 0.45 (95% CI: 0.01 to 0.75) two quarters prior. Deviation from expected pre-pandemic trends began in 2020, but worsened dramatically from 2023 onwards (Additional file 5, Figure S91).

**Conjunctivitis (F70)**

In 2024, F70 (conjunctivitis infectious) exceeded the modelled baseline by 34,643 consultations (95% PI: 31,799 to 37,490)—a relative excess of 59% (95% PI: 51% to 67%) (Figures 2 and S3 and Table S1). Relative excess varied significantly with age, with 15–19 year olds not significantly differing from the modelled baseline and 5–14 year olds having the largest relative excess (146%). Females had a higher relative excess (70%) than males (43%) (Additional file 5, Table S34).

Temporal correlations with COVID-19 community spread were 0.42 (95% CI: -0.03 to 0.73) in the same quarter, 0.65 (95% CI: 0.29 to 0.85) in the prior quarter, and 0.54 (95% CI: 0.13 to 0.79) two quarters prior. Consultations were lower than the modelled baseline in 2020–2021, as expected in 2022, higher than expected in 2023, and much higher than expected in 2024 (Additional file 5, Figure S67).

**Infectious disease other/no obvious source (A78)**

In 2024, A78 (infectious disease other/NOS) exceeded the modelled baseline by 33,556 consultations (95% PI: 26,397 to 40,606)—a relative excess of 81% (95% PI: 54% to 118%) (Figures 2 and S3 and Table S1). Relative excess varied significantly with age, with 5–14 year olds (310%) having the largest relative excess, followed by 20–29 year olds (121%), 15–19 year olds (94%), 30–64 year olds (88%), and 65+ year olds (27%). There was no noticeable variation in relative excess by sex (Additional file 5, Table S14).

Temporal correlations with COVID-19 community spread were 0.35 (95% CI: -0.11 to 0.69) in the same quarter, 0.39 (95% CI: -0.07 to 0.71) in the prior quarter, and 0.53 (95% CI: 0.12 to 0.79) two quarters prior. Deviation from expected pre-pandemic trends began in 2021, but worsened dramatically from 2022 onwards (Additional file 5, Figure S27).
