# Supplementary material for: Excess primary healthcare consultations in Norway in 2024 compared to pre-COVID-19-pandemic baseline trends
Source: Arch Public Health. 2026 Jan 2;84:26. doi: 10.1186/s13690-025-01817-8 (PMC12866491; doi:10.1186/s13690-025-01817-8)
Supplement: Supplementary file 7 — Additional file 7. Detailed alternate theories than COVID-19 supplement. [file 13690_2025_1817_MOESM7_ESM.docx]

**Additional File 7.** Detailed alternate theories than COVID-19 supplement.

Before considering alternative hypotheses, it is important to address the apparent contradiction between declining diagnosis codes for acute COVID-19 and the hypothesis of ongoing SARS-CoV-2 impact. The decline in consultations with direct COVID-19 codes (R991+R992) from 2023 onwards does not represent a true magnitude of reduced transmission but rather changes in testing policy. From 2023, for the majority of the population, the Norwegian Institute of Public Health recommended against testing for COVID-19 when symptomatic [1]. This has led to few being tested for COVID-19, including patients presenting with symptoms of respiratory infection at GP consultations and emergency room visits. This again has led to substantial underreporting despite continued widespread SARS-CoV-2 circulation.

The composition of consultations for respiratory infections has shifted substantially over time. In the early pandemic period (2020–2022), the respiratory infections excess was predominantly acute COVID-19. As shown in Table 1, acute COVID-19 codes (R991+R992) accounted for 70.1%, 53.8%, and 67.5% of all excess consultations in 2020, 2021, and 2022 respectively. However, the pattern changed dramatically in the later pandemic period (2023–2024). By 2024, R991+R992 accounted for 5.1% of excess consultations, yet respiratory infections remained substantially elevated with striking increases in several specific non-COVID conditions: whooping cough (343% relative excess), pneumonia in children aged 5–14 (1,066% relative excess), and cough in the same age group (257% relative excess). This temporal shift suggests the respiratory infection burden now reflects a combination of miscategorized acute COVID-19 cases (since testing was no longer recommended for most of the population from 2023 onwards) and increased susceptibility to other respiratory pathogens. As noted throughout our discussion, distinguishing the relative contributions of these mechanisms is not possible with our data, though the sustained elevation and specific age patterns (particularly in school-aged children) are consistent with documented post-acute sequelae features including immune dysregulation and increased infection susceptibility. Some research has found clear associations between a positive SARS-CoV-2 test and subsequent increased rates of outpatient diagnosis of infectious illnesses [2], while others report no such associations [3]. An alternative hypothesis is that more severe respiratory pathogen outbreaks occurred after COVID-19 mitigations were removed due to immunological debt from reduced pathogen exposure. However, the temporal correlations between respiratory infection increases and COVID-19 community spread waves, combined with the persistence of elevated susceptibility two years after mitigations ended in 2022, suggest factors beyond simple rebound effects are contributing to the observed patterns.

It is not possible to state what proportion of the observed increases are due to acute or post-acute COVID-19. Furthermore, there are other factors that undoubtedly play a role in the observed increases. When considering hypotheses other than COVID-19, alternative hypotheses must account for the fact that 2024 consultation rates for our ten ICPC-2 code combinations exceeded pre-pandemic trend projections. Pre-existing factors cannot explain these increases unless the underlying trends themselves accelerated after 2020 (Figure 5).

A previous study noted an increase in ADHD-medication from 2010–2020 across Scandinavia [4], which is in line with what our study observed from 2010–2019 regarding primary healthcare consultations for hyperkinetic disorder. Some researchers have suggested that the increase in diagnosis and treatment of psychological and cognitive complaints may be related to increased medicalization of ADHD [5] or a widening diagnostic criteria [6]. Crucially, however, our study observed an even larger increase from 2020 onwards that rapidly outpaced pre-pandemic trends (this has also been observed in Denmark [6]), suggesting that either a new risk factor has occurred (e.g. COVID-19) or there has been a further increase of the pre-pandemic causes of the increase (i.e. the rate at which society is medicalizing ADHD has increased significantly since 2020 or the diagnostic criteria has widened significantly since 2020), or both (Figure 5).

An important consideration is whether our findings for psychological complaints reflect COVID-19 impacts versus broader national or global mental health trends [7]. The magnitude and sustained elevation from 2022–2024 suggest an acute acceleration beyond historical trends. That is, our findings therefore are either the result of new risk factors, or increases of the pre-pandemic causes of these worsening trends, or both (Figure 5). While both medicalization [5] and widening diagnostic criteria [6] have been proposed as potential causes, it is unclear if they have increased post-2020. Three primary novel risk factors that have emerged since 2020 are the ongoing large increase in COVID-19 incidence in the population, social disruption due to the pandemic mitigations between 2020–2021, and a cost-of-living crisis between 2022–2023. While studies have found that the acute phase of the pandemic and pandemic mitigations were associated with worsening mental health [8], further research is required to understand if there is an ongoing impact in 2024. Norway’s cost-of-living crisis eased in 2024 [9], however, lingering impacts likely remain. Finally, our findings encompass diverse health impacts beyond mental health, including physical symptoms (fatigue, abdominal pain), infectious diseases (respiratory infections, conjunctivitis), and cognitive symptoms (memory disturbance, hyperkinetic disorder).

When considering the increase in primary healthcare consultations for psychological complaints, a similar increase has been observed for medically certified sick leave [10]. According to the Norwegian Labour and Welfare Administration, the post-2021 increase in medically certified sick leave for psychological complaints resulted from both longer leave durations and more individuals taking such leave [10]. Furthermore, the authors found that sick leave for psychological issues increased across the entire Norwegian population, indicating that there are factors influencing the whole population, not just vulnerable groups, at play [10]. Ultimately, the authors found no support for the hypotheses that the increase in psychological issues is caused by social media, changes in sick leave incentives, internet/screen-time usage, increased work pressure, understaffing, or increase in home office usage [10].

While healthcare digitalization may contribute to increased consultation rates by improving access, it cannot fully explain the observed patterns. If digitalization were the primary driver, we would expect relatively uniform increases across conditions amenable to telemedicine. Instead, we observed markedly differential relative excesses: psychological symptoms (80%+), hyperkinetic disorder (116%), memory disturbance (63%), and fatigue (70%) far exceeded the relative excesses in other telemedicine-suitable conditions such as skin rashes (30%). The mean annual number of consultations per general practitioner also returned to pre-pandemic levels by 2023 [11], indicating that by 2023 e-consultations replaced physical consultations without additional healthcare seeking behavior. These patterns suggest digitalization alone cannot account for the magnitude and specificity of observed increases.

Delayed healthcare, pent-up demand, general pandemic disruption, and economic stress could theoretically contribute to increased primary healthcare utilization. In particular, one hypothesis is that excess consultations during 2020–2022 reflected direct COVID-19 care, while increases in later years represent backlogged healthcare services accumulated during the acute pandemic phase. However, several observations argue against these explanations as fully explaining the observed patterns for these ten ICPC-2 code combinations. First, due to effective mitigation strategies, Norway’s healthcare was not overwhelmed during the acute phase of the pandemic [12]. Second, pent-up demand typically manifests as a temporary spike followed by return to baseline as backlogs are cleared, whereas our data show sustained elevation through 2024 with consultation rates remaining 7.1% above expected levels. Third, the diagnostic specificity of increases—concentrated in conditions compatible with PASC such as fatigue, memory disturbance, and respiratory symptoms—is inconsistent with general patterns of delayed routine or preventive care. Finally, the temporal correlations between increases and SARS-CoV-2 circulation patterns, along with persistence through 2024 despite economic recovery [9], suggest factors beyond pandemic disruption and economic stress are at play.

[1] Fausko L. FHI-overlege om coronatesting: – Jeg gidder ikke. VG 2023. https://www.vg.no/nyheter/i/8JK78r/fhi-om-coronatesting-jeg-gidder-ikke (accessed July 2, 2025).

[2] Files JK, Boppana S, Perez MD, Sarkar S, Lowman KE, Qin K, et al. Sustained cellular immune dysregulation in individuals recovering from SARS-CoV-2 infection. J Clin Invest 2021;131. https://doi.org/10.1172/JCI140491.

[3] Allen AJ, Nguyen N, Lorman V, Maltenfort M, Saleh Mohammad Mosa A, Sekar A, et al. Respiratory and Other Infections Following COVID. Pediatrics 2025;156:e2024068280. https://doi.org/10.1542/peds.2024-068280.

[4] Sørensen AMS, Wesselhöeft R, Andersen JH, Reutfors J, Cesta CE, Furu K, et al. Trends in use of attention deficit hyperactivity disorder medication among children and adolescents in Scandinavia in 2010–2020. Eur Child Adolesc Psychiatry 2023;32:2049–56. https://doi.org/10.1007/s00787-022-02034-2.

[5] Engström I. Explosive Increase in Diagnosis and Treatment of ADHD in Sweden May Be Related to Private Health Providers Offering Fast‐Track, Guaranteed Diagnoses. Acta Paediatr 2025;114:2095–7. https://doi.org/10.1111/apa.70129.

[6] Kildegaard H, Wesselhoeft R, Lund LC, Bliddal M. Post‐pandemic trends in psychotropic medication use in Danish children, adolescents, and young adults. Acta Psychiatr Scand 2024;150:174–7. https://doi.org/10.1111/acps.13719.

[7] Ferrari AJ, Santomauro DF, Aali A, Abate YH, Abbafati C, Abbastabar H, et al. Global incidence, prevalence, years lived with disability (YLDs), disability-adjusted life-years (DALYs), and healthy life expectancy (HALE) for 371 diseases and injuries in 204 countries and territories and 811 subnational locations, 1990–2021: a systematic analysis for the Global Burden of Disease Study 2021. The Lancet 2024;403:2133–61. https://doi.org/10.1016/S0140-6736(24)00757-8.

[8] Lu L, Hannigan LJ, Brandlistuen RE, Nesvåg R, Trogstad L, Magnus P, et al. Mental Distress Among Norwegian Adults During the COVID-19 Pandemic: Predictors in Initial Response and Subsequent Trajectories. Int J Public Health 2023;68:1606164. https://doi.org/10.3389/ijph.2023.1606164.

[9] OECD. OECD Economic Outlook, Volume 2025 Issue 1: Preliminary version. vol. 2025. OECD Publishing; 2025. https://doi.org/10.1787/83363382-en.

[10] Nossen JP, Delalic L. Hvorfor er sykefraværet fortsatt høyt 3–4 år etter starten av pandemien? | Arbeid og velferd. Arb Og Velferd n.d.;2024.

[11] White RA, Zhang C, Valcarcel Salamanca B, Angelsen A, Zakiudin DP, Andries A, et al. Aberrations in medically certified sick leave and primary healthcare consultations in Norway in 2023 compared to pre-COVID-19-pandemic trends. Arch Public Health 2024;82:187. https://doi.org/10.1186/s13690-024-01411-4.

[12] Tu K, Lapadula MC, Apajee J, Bonilla AO, Baste V, Cuba-Fuentes MS, et al. Changes in reasons for visits to primary care after the start of the COVID-19 pandemic: An international comparative study by the International Consortium of Primary Care Big Data Researchers (INTRePID). PLOS Glob Public Health 2024;4:e0003406. https://doi.org/10.1371/journal.pgph.0003406.
